# On Modern Progress in Ophthalmic Medicine and Surgery

**Published:** 1895-07-20

**Authors:** Robert Brudenell Carter

**Affiliations:** Consulting Ophthalmic Surgeon to St. George's Hospital


### On Modern Progress in Ophthalmic Medicine and Surgery.

By Robert Brudenell Carter, F.R.C.S., Consulting Ophthalmic Surgeon to St. George's Hospital.

### DISEASES OF THE CORNEA.

(*Continued from page* 197.)

### Conical Cornea.

Besides the changes wrought by manifest morbid processes, the cornea is liable to a deformity of shape which, in its earlier stages, may easily escape observation, but which is liable to increase until it becomes of grave importance as a cause of visual defect. This deformity is called conical cornea, or sometimes "keratoconus," or merely "conicity," and it depends upon a yielding and bulging forward of the central portion, so that the whole cornea assumes more or less the shape of a pyramid or of a cone. When pronounced, the aspect of conical cornea is not to be mistaken. As seen from the front, the centre of the eye appears to be of an unnatural brightness or increased refractive index, and almost glitters, in a way which has often been compared to the effect of a drop of dew upon the petal of a flower. When the eye is looked at in profile the altered curve of its outline becomes conspicuous. In an earlier stage conicity may be recognised by allowing the light from an ophthalmoscopic mirror to play to and fro over its surface, while the observer looks through the mirror aperture. An irregular shadow is then seen to fall upon the illuminated pupil, and to alter its position as that of the light varies. This test was first employed by the late Sir William Bowman, and the principle has since been extended to the investigation of refractive errors in general. At a later stage, when the prominence is so considerable as to be deprived of shelter from the lids, the centre of the cornea often becomes roughened or opaque.

The effect of conicity upon vision is exceedingly detrimental. The length of the antero-posterior axis of the eyeball being increased, there is, of course, a corresponding degree of myopia; but, from the shape of the cone, the myopia opposite the centre of the pupil is greater than that of the parts or zones around the centre, so that no single glass will afford useful correction of the faulty curvature as a whole. The outlines of the "cone," moreover, are seldom symmetrical, so that, even when still transparent, the cornea becomes a distorting medium. When its centre has been rendered opaque by friction and exposure, the condition of the patient differs but little from blindness.

Conical cornea is usually an affection of early adult life, and is very much more common in females than in males, in whom, on the other hand, it may occur at or about puberty. The ordinary sufferer from it is an anæmic or somewhat overgrown young woman, much of the same type as the ordinary subject of gastric ulcer; but I have met with a few examples in persons of apparently sound health.

Prior to the commencement of central opacity, no morbid process can be discovered in the cornea when examined in the living subject; and the pathology of the affection remained unknown until eyes were obtained for examination from persons dying of some other disease during the progress of the change. The conicity was then found to be a result of simple atrophy of the cornea, first and chiefly affecting the central parts, as those most remote from the sources of nutrition, the thinned and weakened membrane yielding to intra-ocular pressure and to the traction of the extrinsic ocular muscles, and becoming stretched and altered in shape accordingly. A section made through the apex of the cone shows nothing but central thinning of the cornea. How far this may be due to an impaired power of absorbing the materials of nutrition, and how far to some change in the nerves by which the nutrition is controlled, is at present unknown.

At a period not very remote, conical cornea was regarded as incurable, and the first attempts at treatment were of a very remarkable character. The alteration of shape was probably attributed to over-secretion within the eye, for it was gravely suggested that the affection might be cured by what was called the "emeto-purgative treatment," under which the unfortunate patient was condemned to swallow an emetic every morning, and a purgative every night, and to continue this for the space of one year. It now seems hardly credible that any doctor could be found to prescribe such a course, or any patient to submit to it; but it was prescribed and submitted to, and persons were found who believed that it was beneficial. With this knowledge, it is impossible to wonder that cures are attributed to quack medicines, or that persons outside Bedlam believe in the efficacy of the various nostrums which are offered for sale to the credulous. I have myself met with a bad case of conicity in an elderly gentleman who had undergone the "emeto-purgative treatment" in his youth, and who told me that he had never recovered from the weakness which it left behind. I even remember a controversy in the medical journals of the time at which it was in vogue, as to who was really entitled to claim the merit of having originated it.

The next step in the history rested upon a somewhat better foundation. In consequence of the irregularity of the so-called "cone," there is usually a considerable amount of astigmatism, with the result that vision is improved by looking through a narrow slit placed before some one meridian of the cornea, and perhaps supplemented by a lens. Machines in the nature of spectacles were made to carry such slits before the eyes, but they were very unsightly, and the progressive character of the disorder tended soon to impair or to destroy their usefulness. Nevertheless, both the late Sir William Bowman and the late Mr. Critchett thought it might be possible to utilise the slit, and for this purpose to convert the pupil itself from a circular into a slit-like opening. The meridian of best vision being ascertained, operations were performed upon the iris for the purpose of incarcerating it at each extremity of this meridian. The results were entirely disappointing. In the first place, the iris did not possess sufficient rigidity to enable the pupil to retain any form which could be described as "slit-like," and although it became an ellipse with its major axis in the desired direction, it usually retained quite a considerable diameter in the opposite one. In the second place, the incarceration itself became a source of danger to the eye, and led to attacks of iritis, which had a tendency to become recurrent and ultimately destructive. It was manifectly necessary to seek for a remedy on some other lines than these; and we are indebted to the late Albrecht von Græfe for the suggestion which has led to ultimate success.

